# ADAR Therapeutics as a New Tool for Personalized Medicine

**DOI:** 10.3390/genes16010077

**Published:** 2025-01-11

**Authors:** Matteo Bertoli, Luca La Via, Alessandro Barbon

**Affiliations:** 1 Department of Molecular and Translational Medicine, University of Brescia, 25123 Brescia, Italy; matteo.bertoli@unibs.it (M.B.); luca.lavia@unibs.it (L.L.V.); 2 Consorzio Interuniversitario per le Biotecnologie, 34121 Trieste, Italy; 3 Center for Colloid and Surface Science (CSGI), Via della Lastruccia 3, Sesto Fiorentino, 50019 Firenze, Italy

**Keywords:** RNA editing, ADAR1, ADAR2, RNA therapeutics, SDRE

## Abstract

In the field of RNA therapy, innovative approaches based on adenosine deaminases acting on RNA (ADAR)-mediated site-directed RNA editing (SDRE) have been established, providing an exciting opportunity for RNA therapeutics. ADAR1 and ADAR2 enzymes are accountable for the predominant form of RNA editing in humans, which involves the hydrolytic deamination of adenosine (A) to inosine (I). This inosine is subsequently interpreted as guanosine (G) by the translational and splicing machinery because of their structural similarity. Intriguingly, the novel SDRE system leverages this recoding ability of ADAR proteins to correct the pathogenic G to A nucleotide mutations through a short, engineered guide RNA (gRNA). Thus, ADAR-mediated SDRE is emerging as a powerful tool to manipulate the genetic information at the RNA level and correct disease-causing mutations without causing damage to the genome. Further it is emerging as a new instrument for personalized medicine, since treatments can be tailored to the unique genetic mutations present in an individual patient. In this short review, we aimed to described the main approached bases on ADARs activity, highlighting their advantages and disadvantages.

## 1. RNA Editing

RNA editing is an epitranscriptomic process by which RNA molecules are enzymatically modified. This phenomenon diversifies the transcriptome and adds an additional layer of gene regulation [1,2].

The most common form of RNA editing in mammals involves the conversion of adenosine (A) to inosine (I), a process mediated by enzymes known as ADARs (adenosine deaminases acting on RNA) [2]. Another form of RNA editing involves the conversion of cytosine (C) to uridine (U), catalyzed by APOBEC (apolipoprotein B mRNA editing enzyme, catalytic polypeptide) enzymes [3]. Unlike A-to-I editing, C-to-U editing is less common but equally important, particularly in certain tissues and developmental stages. A-to-I RNA editing can affect various aspects of RNA metabolism, including splicing, stability, transport, and translation efficiency [4,5,6,7]. Moreover, RNA-editing events can change the coding sequence of mRNAs, potentially leading to the production of proteins with altered functions, which can have significant implications for cellular processes and organismal development [8]; dysregulation of RNA editing has been implicated in various diseases, including neurological disorders and cancer [8,9,10].

Research into RNA editing is continually expanding, revealing its complexities and uncovering new implications for understanding gene-expression regulation and potential therapeutic interventions. For instance, recent studies have uncovered the extensive occurrence of A-to-I editing in the human brain, highlighting its importance in neural plasticity and adaptability [11], where it contributes to the diversity of the ion channels, receptors, and other proteins involved in neurotransmission [12]. Furthermore, RNA editing has been shown to play a role in the immune response, affecting the expression and function of cytokines and other immune-related proteins [13].

RNA editing also interacts with other RNA-processing events, such as alternative splicing. Editing events can create or destroy splice sites, leading to the inclusion or exclusion of exons in the mature transcript. This interplay adds another layer of complexity to the regulation of gene expression. In addition, RNA editing can influence the stability and localization of mRNAs. Edited RNAs can evade recognition by nucleases, thereby increasing their stability [14]. The localization of mRNAs within the cell can also be affected by editing, as it can alter the binding affinity of RNA-binding proteins and their interactions with the cytoskeleton [7,15].

The functional impact of RNA editing extends beyond the coding regions of mRNAs. Non-coding RNAs, such as microRNAs (miRNAs) and long non-coding RNAs (lncRNAs), are also targets of RNA editing. Editing of miRNA precursors can influence their processing and the targeting of mature miRNAs to their mRNA targets [16]. Similarly, editing of lncRNAs can affect their interactions with other molecules and their roles in regulating gene expression [17].

Understanding the physiological role of RNA editing is crucial for developing potential therapeutic strategies. Targeting the RNA-editing machinery or the edited sites themselves could provide novel approaches for treating diseases associated with dysregulated RNA-editing levels. For example, correcting defective RNA editing in specific genes could restore normal protein function and ameliorate disease symptoms [10].

## 2. Structural Characteristics of ADAR Enzymes

ADARs are pivotal enzymes in the post-transcriptional-modification landscape, responsible for converting adenosine (A) to inosine (I) within RNA molecules [1,18]. The importance of ADARs extends across various biological functions, including the regulation of gene expression, nervous-system functionality, and immune-response modulation. The ADAR enzyme was first described in the late 1980s [19].

ADARs are characterized by a modular structure comprising several distinct domains that coordinate to facilitate RNA binding and editing. The key structural features include the following:

- Double-stranded RNA-Binding Domains (dsRBDs): ADAR enzymes generally have one or more dsRBDs, which are essential for identifying and attaching to double-stranded RNA (dsRNA) substrates. These domains are well-preserved across species and help the enzyme locate and bind to its specific RNA targets [2].
- Deaminase Domain: The deaminase domain serves as the central catalytic hub of ADAR enzymes, tasked with converting adenosine to inosine through hydrolytic deamination [7].
- Z-DNA Binding Domain (ZBD): The ZBD is capable of attaching to Z-DNA, which is a left-handed DNA helix. While the exact function of this domain is not entirely understood, it is hypothesized that it plays a role in detecting cellular stress [17].

In mammals, two active ADAR enzymes, ADAR1 and ADAR2, have been discovered: ADAR1, which contains three dsRBDs, whereas ADAR2 has two [1]. ADAR1 is expressed into two forms: An interferon-inducible p150 protein is mainly involved in antiviral defense and localized in the cytoplasm. A shorter p110, a constitutively expressed isoform of ADAR1, results in a protein that localizes mainly to the nucleus [20,21]. Conversely, ADAR2 is primarily found in the nervous system, localizing to the nucleus [22]. An additional ADAR enzyme, ADAR3, which seems to exhibit no catalytic activity, has been described. However, it may compete with ADAR1 and ADAR2 for RNA-binding sites, thereby potentially modifying the RNA-editing landscape through this competitive mechanism [23]. Very recently, ADAR3 has been suggested to negatively regulate neuronal differentiation by regulating mRNA stability and translation in an editing independent manner [24]. The mechanism of A-to-I RNA editing is a multistep process orchestrated by the interaction of different structural domains within ADAR enzymes. Initially, the dsRBDs of ADAR enzymes identify and attach to specific regions of dsRNA, which often exhibit imperfect base pairing, resulting in loops and bulges that allow the enzyme to access adenosine residues [17,25]. Once bound, the deaminase domain positions the adenosine within its active site. With the help of a conserved zinc ion and catalytic residues, this domain removes an amino group from the adenosine, converting it to inosine through hydrolytic deamination. Following this conversion, the edited RNA adopts a structure where inosine pairs with cytidine, mimicking guanosine, which can alter the RNA’s secondary structure and influence processes like splicing, translation, and stability [10].

## 3. Therapeutic Potential of ADAR Enzymes

Approximately 60% of genetic disorders are caused from single nucleotide polymorphisms, and RNA editing has the potential to rectify the prevalent G-to-A alterations, which represent nearly 28% of these SNPs [26,27]. These mutations can cause amino acid substitutions or modify stop codons within coding regions. The effects of these mutations can be extensive, contributing to the development of tumors, genetic diseases, and metabolic disorders.

The range of treatments available for genetic disorders is rapidly expanding, with numerous clinical trials in progress focusing on gene therapy and gene editing. Traditional treatments, however, are effective for only 10% of these disorders [27,28].

Gene editing seeks to alter or substitute flawed genetic material through methods like transcription activator-like effector nucleases (TALENs), zinc-finger nucleases (ZFNs), and the highly adaptable CRISPR-Cas9 technology. These advancements are revolutionizing genetic research and therapeutic applications. Although genome editing holds significant promise, it encounters substantial challenges. These include the potential for causing irreversible genomic changes and the difficulty of applying it to postmitotic cells, which do not have reliable DNA-repair mechanisms. Moreover, ensuring the safe and efficient delivery of the required components to target cells remains a major hurdle. Addressing these issues is essential for the wider adoption of genome-editing techniques in clinical practice [29].

## 4. Site-Directed RNA Editing

**Figure 1:**
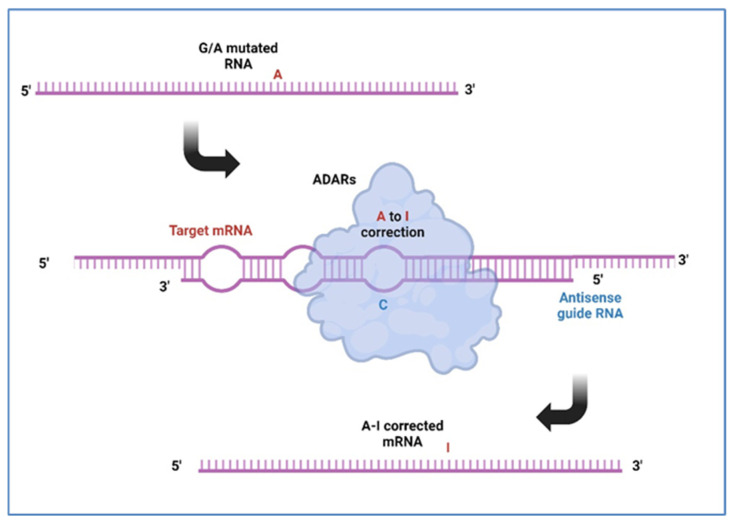
Specific antisense guide RNA might recruit ADAR enzymes to the mutated target mRNA inducing site-direct RNA editing thus generating a corrected mRNA. A: adenosine; I: inosine; C: cytidine (created with BioRender.com).

Very recently, innovative approaches based on ADAR-mediated site-directed RNA editing (SDRE) have been established, thus providing an exciting opportunity for RNA therapeutics of gene disorders [30,31]. Programmable RNA editing, leveraging ADAR enzymes, presents a groundbreaking approach to treat genetic disorders at the RNA level, bypassing the potential risks associated with permanent DNA modifications (Figure 1). By designing guide RNAs that direct ADAR enzymes to specific RNA sequences, treatments can be tailored to the unique genetic mutations present in an individual patient. SDRE might be considered as a new tool for personalized medicine, allowing for highly specific and individualized treatment of genetic diseases.

Specifically, the SDRE system can use either exogenous or endogenous ADAR as catalytically active deamination effectors. Both approaches require the synthesis and delivery of a guide RNA (gRNA) that is antisense to the target sequence and introduces an A-C mismatch at the A target (mutated nucleotide).

## 5. Exogenous ADAR System

### 5.1. SNAP–ADAR

The SNAP–ADAR technology represents a highly adaptable strategy for SDRE, effectively applied in a different cellular system (Table 1). This approach merges the deaminase domain of ADAR with the SNAP enzyme, employing a modified guide RNA (gRNA) that is linked to a O6-benzyl-guanine [32,33]. The SNAP–ADAR complex linked to the gRNA will be guided to the mRNA target to induce site-directed editing. Importantly, the SNAP–ADAR technique can reach a remarkable editing efficiency, especially when utilizing a hyperactive mutant variant of ADAR2. In addition to its high efficiency, the versatility of the SNAP–ADAR system makes it a promising tool for various therapeutic applications, potentially revolutionizing the treatment of genetic disorders by providing precise and controlled RNA-editing capabilities [34]. For instance, the SNAP–ADAR system has achieved an editing efficiency of up to 90%, making it a promising solution for various therapeutic applications [33].

Very recently, the SNAP–ADAR tool has been applied to interfere with cellular signal transduction, modifying amino acids that may be subject to post-translational modification [35]. Given that the ADAR fusion is derived from a human origin, the probability of eliciting an immunogenic response is considerably low [36].

**Table 1:** Exogenous ADAR-based systems.

System	Mechanism of Action	References
SNAP–ADAR	This approach merges the deaminase domain of ADAR with the SNAP enzyme, employing a modified guide RNA (gRNA) that is nuclease-resistant and linked to O6-benzyl-guanine (BG).	[32,33]
	Level of correction achieved up to ~90%.	
REPAIR	This method combines the deaminase domain of ADAR2 with a Cas13b orthologue creating a programmable RNA editor and allowing precise A-to-I RNA editing.	[37]
	Level of correction achieved up to ~30%(REPAIRv1)/27%(REPAIRv2).	
RESCUE	This technique relies on engineered mutations in the ADAR^DD^ enzyme and facilitates both A-to-I and C-to-U modifications, increasing the number of potentially correctable mutations.	[36,38]
	Level of correction achieved up to ~42%.	

### 5.2. CRISPR-Based Tool

Recent advancements in CRISPR (clustered regularly interspaced short palindromic repeats)-based genome-editing methodologies have attracted considerable scientific attention for their characteristics and extensive applicability across various biological systems [36]. The discovery of Cas13 has broadened the scope of CRISPR technology. Unlike the more commonly known Cas9, which targets DNA, Cas13 is unique in its ability to target RNA (Table 1) [37].

#### 5.2.1. REPAIR

The REPAIR technology (RNA Editing for Programmable A-to-I Replacement) is a cutting-edge approach for therapeutic applications [37]. It leverages the enzyme ADAR2 fused to a Cas13b orthologue, allowing precise A-to-I RNA editing. The guide RNA (gRNA) employed in this system includes an A-C mismatch at the adenosine site targeted for editing. A spacer sequence distinguishes this region from a direct repeat segment that recruits Cas13b to the target sequence (Table 1) [37]. A REPAIRv2 has been developed by reducing the size of Cas13 which minimizes off-target editing and has been used to edit CFTR mRNA with premature stop codons [39].

#### 5.2.2. RESCUE

RESCUE (RNA Editing for Specific C to U Exchange) is the initial tool developed for C-to-U RNA editing [38]. This technique relies on engineered mutations in the deamination domain of the ADAR (ADAR^DD^) enzyme, enabling the deamination of cytosines. This ability facilitates both A-to-I and C-to-U modifications, vastly broadening the range of mutations that can be effectively corrected. The engineered enzyme is conjugated to an inactivated Cas13 orthologue, functioning similarly to the REPAIR system. The main issue encountered with this platform is the increase in off-target effects due to its ability to edit not only adenosines but also cytosines (Table 1) [36].

## 6. Endogenous ADAR System

Recent advancements in RNA-editing technologies have leveraged endogenous ADAR enzymes to achieve precise and efficient RNA modifications, reducing the risk of immunogenic responses (Table 2). This approach involves the use of guide RNAs (gRNAs) that specifically direct endogenous ADARs to the target site. These gRNAs hybridize with the target RNA, creating mismatches that ADARs recognize and edit. By coupling ADARs with these gRNAs, researchers can achieve targeted RNA editing with high specificity and minimal off-target effects (Table 3) [28,40].

### 6.1. LEAPER

LEAPER (Leveraging Endogenous ADAR for Programmable Editing of RNA) is a technique that utilizes long antisense RNAs, up to 200 nucleotides, to create dsRNA to recruit and direct endogenous ADAR enzymes to specific adenosines within RNA targets [41]. Similar to other methods, the guide RNA (gRNA) features a single A-C mismatch at the target location to facilitate precise editing (Table 2) [28]. It has been shown that loops of 8 nt or more help to add selectivity of ADAR binding to dsRNA, and A-G mismatch avoids or decreases bystander editing on nontarget adenosine [47]. Further, the use of adeno-associated virus (AAV) vectors for delivering circular ADAR-recruiting guide RNAs (arRNAs) ensures high efficiency and sustained editing, highlighting the therapeutic potential of a developed version called LEAPER 2.0 [48]. This method has shown substantial promise in correcting genetic mutations associated with diseases. In the study conducted by Yi et al., researchers demonstrated the efficacy of this approach in correcting nonsense mutations in models of Hurler syndrome [48].

This technology can achieve up to 50% editing efficiency on endogenous targets while maintaining a low rate of off-target edits [28]. The unengineered nature of the RNA without chemical alterations permits its delivery. This makes the RNA guide easier to introduce into various cell types [36,42].

### 6.2. RESTORE

RESTORE (Recruiting Endogenous ADAR to Specific Transcripts for Oligonucleotide-Mediated RNA Editing) is a technique that takes advantage of the body’s natural RNA-editing processes. It employs short, chemically modified antisense oligonucleotides, ranging from 20 to 40 nucleotides in length, to direct endogenous ADAR enzymes to specific target sites. These guide RNAs (gRNAs) consist of two key domains: an invariant R/G ADAR recruitment domain, which ensures that the endogenous ADAR enzyme is accurately directed to the double-stranded RNA (dsRNA), and a programmable specificity domain, which determines the binding specificity to the target mRNA [42,43]. This dual-domain configuration allows for precise and efficient RNA editing by utilizing the body’s inherent mechanisms, leading to targeted mRNA modifications with high specificity and minimized off-target effects [42]. RESTORE has shown promise in correcting mutations associated with various genetic disorders, presenting a versatile and effective tool for RNA editing (Table 2) [36].

### 6.3. CLUSTER

CLUSTER (Clustered ADAR-Recruiting Guide RNAs for Efficient RNA Editing) represents a significant advancement in RNA-editing technology, enabling precise and efficient RNA editing by leveraging endogenous ADAR enzymes in vivo [44]. This innovative approach builds on previous R/G-gRNA designs by incorporating a cluster of single-stranded RS regions, which bind to various regions of the target mRNA, enhancing the binding strength and specificity. In contrast to the LEAPER method, which extends the specificity domain, the RS elements independently bind to various distal regions of the target mRNA and to each other [44]. This technique involves using multiple guide RNAs that bind to adjacent regions of the target RNA, creating a clustered editing site. The clustered guides increase the local concentration of ADAR enzymes at the target site, thereby boosting the efficiency and accuracy of A-to-I editing. CLUSTER has shown promise in preclinical studies for its ability to achieve high editing efficiency with minimal off-target effects (Table 2) [44].

In cell culture, CLUSTER gRNAs have demonstrated on-target editing of endogenous transcripts with yields of up to 45% without bystander editing. In vivo studies have shown that CLUSTER gRNAs delivered to mouse livers via hydrodynamic tail vein injection achieved editing rates of up to 10% [44].

This method is particularly useful for applications requiring extensive and precise RNA modifications, such as correcting complex mutations or editing multiple sites within a single transcript.

### 6.4. Circular ADAR-Recruiting Guide RNAs (cadRNAs)

Circular ADAR-recruiting guide RNAs (cadRNAs) represent a novel advancement in RNA-editing technology. These cadRNAs are designed to harness the body’s endogenous ADAR enzymes for programmable A-to-I RNA editing without the need for codelivery of exogenous proteins. CadRNAs can be genetically encoded and expressed within cells to attract ADARs enzymes, leading to precise and efficient RNA editing [45,46]. Litke et al. introduce the Tornado system (twister-optimized RNA for durable overexpression), which enables rapid circularization of RNA thanks to the addition of ribozymes at the 5′ and 3′ ends of the ASO, resulting in RNAs with enhanced stability and significantly higher expression levels [46]. Katrekar and colleagues have demonstrated the high efficiency of this system also to produce antisense oligonucleotides capable of recruiting ADAR enzymes.

One of the key benefits of cadRNAs is their high stability and durability, which results in robust RNA editing across multiple sites and cell lines. This technology has shown significant results, achieving up to 50% editing efficiency on endogenous targets. Additionally, cadRNAs can be delivered via various methods, including viral vectors and in vitro-transcribed RNA molecules, making them versatile for different therapeutic applications (Table 2) [45].

In vivo studies have demonstrated the potential of cadRNAs for therapeutic use, with successful RNA editing observed in animal models. For example, cadRNAs delivered via adeno-associated viruses (AAVs) achieved 53% RNA editing of the mPCSK9 transcript in mouse livers and 12% correction of a nonsense mutation in a mouse model of mucopolysaccharidosis type I-Hurler syndrome [45].

Overall, cadRNAs offer a promising approach for precise and efficient RNA editing, with applications in gene therapy and protein modulation. Their ability to leverage endogenous ADAR enzymes and their high specificity make them a valuable tool in the field of RNA therapeutics [45].

Recent research has highlighted its feasibility and therapeutic potential, particularly for conditions like inherited retinal diseases (IRDs) and genetic disorders such as Hurler syndrome [41,48]. The study conducted by Bellingrath et al. explores the therapeutic use of ADAR enzymes to correct pathogenic mutations causing IRDs. By deploying circular guide RNAs that recruit ADARs, researchers have demonstrated successful editing of disease-causing mutations in retinal cells, enhancing both the efficiency and specificity of RNA editing [41].

## 7. Clinical Trials

As RNA editing moves closer to clinical implementation, it holds promise for treating a wide array of genetic disorders with unprecedented specificity and efficiency, paving the way for new therapeutic paradigms. Among the promising candidates are WVE-006, KRRO-110, AX-1412, and AX-0810, each targeting different diseases through innovative mechanisms [49]:

- WVE-006, developed by Wave Life Sciences (https://wavelifesciences.com/), use short chemically modified oligonucleotides called AIMers that elicit efficient and specific A-to-I editing of endogenous transcripts, which have already been tested in healthy volunteers and is soon to be trialed in patients with α-1 antitrypsin deficiency (AATD) [50,51], (https://clinicaltrials.gov/search?intr=WVE-006, accessed on 2 January 2025).
- KRRO-110, developed by Korro-Bio (https://www.korrobio.com/), targets AATD; their approach involves co-opting endogenous ADAR via a proprietary, engineered oligonucleotide (chemically modified RNA called CHORD or Customized High-fidelity Oligonucleotides for RNA Deamination) to introduce precise edits. KRRO-110 will be tested in both healthy adult participants and in patients (https://clinicaltrials.gov/search?intr=KRRO-110, accessed on 2 January 2025).
- AX-1412, developed by ProQR (https://www.proqr.com/), with the proprietary Axiomer™ technology is based on short strands of synthetic RNA, called editing oligonucleotides or EONs, that enhance ADAR binding on specific RNA target [52]. It targets cardiovascular diseases by introducing a protective variant into the B4GALT1 RNA. The drug aims to reduce the residual risk of developing cardiovascular diseases by modulating gene expression.
- AX-0810, also developed by ProQR, will be tested as a therapeutic approach to lower bile acid reuptake in cholestatic diseases by modulating the NTCP (Na-taurocholate transporting polypeptide) gene [53].

Further, other companies, such as ADARx, Vico therapeutics, and AIRNA, are developing their own drugs with patented technologies, showing the increasing attention of the biotechnologies companies on ADAR therapeutics.

## 8. Conclusions

Programmable RNA editing leveraging ADAR enzymes represents a groundbreaking approach to treat genetic disorders at the RNA level, bypassing the potential risks associated with permanent DNA modifications (Table 4). Recent research has highlighted its feasibility and therapeutic potential, particularly for conditions like inherited retinal diseases (IRDs) and genetic disorders such as Hurler syndrome [41,48].

This technology not only showcases the therapeutic potential of RNA editing but also represents a safer profile due to the transient nature of RNA edits, making it a compelling alternative to traditional gene therapy.

Overall, site-directed RNA editing using ADAR enzymes offers a promising pathway for developing new gene therapies. Continued research and development in this field could revolutionize the treatment of genetic disorders, providing safer and more targeted RNA-level interventions. As RNA-editing technology moves closer to clinical implementation, it holds the promise of treating a wide array of genetic disorders with unprecedented specificity and efficiency, paving the way for new therapeutic paradigms.

## Figures and Tables

**Table 2:** Endogenous ADAR-based systems.

System	Mechanism of Action	References
LEAPER	The technique utilizes long antisense RNAs to create dsRNA to recruit and direct endogenous ADAR enzymes to specific adenosines within RNA targets.	[28,36,41]
	Level of correction achieved up to ~50%.	
RESTORE	Th guide RNAs (gRNAs) have two main parts: a fixed R/G ADAR recruitment domain that directs the ADAR enzyme to the double-stranded RNA (dsRNA) and a programmable domain that binds specifically to the target mRNA.	[36,42,43]
	Level of correction achieved up to ~75–85%.	
CLUSTER	This approach builds on previous R/G-gRNA designs by incorporating a cluster of single-stranded RS elements, which bind to various regions of the target mRNA, enhancing ADAR binding strength and specificity.	[36,42,44]
	Level of correction achieved up to ~45%	
CadRNAs	Based on the use of ribozymes which enables rapid circularization of RNA, CadRNAs have demonstrated enhanced stability and significantly higher expression levels.	[45,46]
	Level of correction achieved up to ~50%.	

**Table 3:** Advantages and disadvantages of ADAR-mediated systems.

System	Advantages and Disadvantages
Endogenous System	Advantages: Endogenous methods offer the advantage of significantly reducing the risk of immune responses by introducing only an antisense oligonucleotide capable of recruiting endogenous ADAR enzymes.
	Disadvantages: Endogenous methods have the disadvantage of having a potential lower efficiency compared to exogenous methods. Secondly, ADAR enzymes are not ubiquitously expressed in all tissues, which limits their applicability.
Exogenous System	Advantages: By incorporating the deaminase domains of ADAR enzymes, exogenous methods demonstrate great applicability and can achieve potentially higher RNA-editing levels.
	Disadvantages: With the exogenous methods, there is a higher likelihood of immune responses and off-target editing.

**Table 4:** Innovations and challenges of site-directed RNA editing.

Innovations of Site-Directed RNA Editing:
**No DNA Modification**: RNA editing allows for the correction of genetic mutations at the RNA level without altering the underlying DNA sequence, avoiding some of the ethical concerns and risks associated with DNA editing.
**Reversibility**: Since RNA editing is transient, it does not have permanent effects, which can be a safer option in certain therapeutic contexts.
**A Targeted Therapy**: It offers a highly specific way to modify gene expression or correct RNA defects in particular tissues or cells, which could lead to more personalized treatments.
**Challenges of Site-Directed RNA Editing:**
**Delivery**: Efficiently delivering the RNA-editing tools (like CRISPR-Cas13 or ADAR enzymes) to the right cells in the body is still a challenge.
**Off-Target Effects**: While RNA editing is generally considered more transient than DNA editing, it still carries the risk of unintended modifications, leading to potential side effects.
**Efficiency**: Achieving efficient and accurate editing in living cells, especially at high precision, is an area of ongoing research.

## Data Availability

No new data were created or analyzed in this study.
